# Future paradigms for precision oncology

**DOI:** 10.18632/oncotarget.9488

**Published:** 2016-05-19

**Authors:** Giannoula Lakka Klement, Knarik Arkun, Dalibor Valik, Tina Roffidal, Ali Hashemi, Christos Klement, Paolo Carmassi, Edward Rietman, Ondrej Slaby, Pavel Mazanek, Peter Mudry, Gabor Kovacs, Csongor Kiss, Koen Norga, Dobrin Konstantinov, Nicolas André, Irene Slavc, Henk van Den Berg, Alexandra Kolenova, Leos Kren, Jiri Tuma, Jarmila Skotakova, Jaroslav Sterba

**Affiliations:** ^1^ Department of Pediatric Hematology/Oncology, Floating Hospital for Children at Tufts Medical Center, Boston, MA, USA; ^2^ Department of Cell, Molecular and Developmental Biology, Sackler School of Graduate Biomedical Sciences, Tufts University, Boston, MA, USA; ^3^ Department of Pathology, Tufts Medical Center, Boston, MA, US; ^4^ Department of Paediatric Oncology, University Hospital Brno, Brno, Czech Republic; ^5^ Regional Center for Applied Molecular Biology, RECAMO, Brno, Czech Republic; ^6^ CSTS Health Care®, Toronto, Canada; ^7^ Computer Science Department, University of Massachusetts, Amherst, MA, USA; ^8^ Central European Institute of Technology, Masaryk University, Brno, Czech Republic; ^9^ 2nd Department of Pediatrics, Semmelweis University, Budapest, Hungary; ^10^ Department of Pediatric Hematology-Oncology, Institute of Pediatrics, Faculty of Medicine, University of Debrecen, Debrecen, Hungary; ^11^ Antwerp University Hospital, Edegem, Belgium; ^12^ Specialized Children's Oncohematology Hospital, Sofia, Bulgaria; ^13^ Department of Pediatric Hematology and Oncology, AP-HM, Marseille, France; ^14^ UMR S_911 CRO2 Aix Marseille Université, Marseille, France; ^15^ Department of Pediatrics, Medical University of Vienna, Vienna, Austria; ^16^ Department of Pediatric Oncology, Emma Children Hospital Academic Medical Centre, University of Amsterdam, Amsterdam, The Netherlands; ^17^ Department of Pediatric Oncology, Comenius University, Bratislava, Slovakia; ^18^ Department of Pathology, University Hospital Brno, Brno, Czech Republic; ^19^ Faculty of Medicine, Masaryk University, Brno, Czech Republic; ^20^ Department of Pediatric Surgery, University Hospital Brno, Brno, Czech Republic; ^21^ ICRC St. Anna University Hospital Brno, Brno, Czech Republic

**Keywords:** precision medicine, targeted therapy, genomics, metronomic chemotherapy

## Abstract

Research has exposed cancer to be a heterogeneous disease with a high degree of inter-tumoral and intra-tumoral variability. Individual tumors have unique profiles, and these molecular signatures make the use of traditional histology-based treatments problematic. The conventional diagnostic categories, while necessary for care, thwart the use of molecular information for treatment as molecular characteristics cross tissue types.

This is compounded by the struggle to keep abreast the scientific advances made in all fields of science, and by the enormous challenge to organize, cross-reference, and apply molecular data for patient benefit. In order to supplement the site-specific, histology-driven diagnosis with genomic, proteomic and metabolomics information, a paradigm shift in diagnosis and treatment of patients is required.

While most physicians are open and keen to use the emerging data for therapy, even those versed in molecular therapeutics are overwhelmed with the amount of available data. It is not surprising that even though The Human Genome Project was completed thirteen years ago, our patients have not benefited from the information. Physicians cannot, and should not be asked to process the gigabytes of genomic and proteomic information on their own in order to provide patients with safe therapies. The following consensus summary identifies the needed for practice changes, proposes potential solutions to the present crisis of informational overload, suggests ways of providing physicians with the tools necessary for interpreting patient specific molecular profiles, and facilitates the implementation of quantitative precision medicine. It also provides two case studies where this approach has been used.

## INTRODUCTION

The conventional approaches to cancer therapy have been until very recently based on eradicating cancer cells by three modalities - surgery, radiation and chemotherapy. While this approach improved outcomes for children with acute lymphoblastic leukemia where survival rose from 20% in the 1950's to about 95% now, it was much less effective in solid tumors and adult leukemias. In these more genetically complex cancers, some modest initial improvements in survival rates were achieved, but even those modest gains have been stagnating since the late 90's. Many different reasons contribute to the treatment resistance of solid tumors and adult leukemias, but chiefly among those are: 1. the genomic complexity and heterogeneity of these entities, and 2. the protective effect of the host / tumor microenvironment.[1, 2] Novel, molecularly-based treatment modalities target not only tumor cells, but also the tumor cell-induced changes in the tumor microenvironment. In addition to those agents directed against tumor cell epitopes and receptor tyrosine kinases, there are monoclonal antibodies directed against endothelial growth factors and receptors, inflammatory cells and immune surveillance cells. All of those can be combined to correct the tumor/microenvironment interaction, and not only sensitize to existing therapies but to effectively target the developmental end-stage characteristics of tumorigenesis.

The term biologic agent is therefore quite broad. It should be considered synonymous with “biological response modifiers”, “targeted agents” or “molecularly-guided therapies”, as well as with other terms used in the broader scientific literature to describe agents that target an otherwise physiological biological events “hijacked” by the tumor for growth benefit. The physiological mechanisms used by tumor cells for survival, i.e. inflammation, angiogenesis, immune system and regenerative pathways, have not been considered as targets in the past, even though wide-ranging spectrum of agents exists for their modulation. They include inhibitors of growth factor pathways, angiogenesis inhibitors, enhancers of pro-apoptotic signals, immune response modifiers, adhesion inhibitors, proteasome inhibitors, signal transduction inhibitors and any other agents targeting a defined biological process in the cancer tissues.

Unfortunately, while all these new insights have come to the forefront of cancer science, their implementation to clinical practice has been quite slow. The understanding that cancer-specific biology may be less dependent on the tissue of origin, and more dependent on a genomic (molecular) signatures, represents a paradigm shift in thinking. This new definition accepts cancer not as foreign tissue, but rather as a natural consequence of lifelong accumulation of molecular alterations, lending credence to therapeutic approach that considers cancer a chronic disease. Unlike the present goal of cancer eradication in a manner similar to antibacterial therapy; scientists now accept that cancer may be managed as a lingering chronic illness influenced by the inflammatory, immune and angiogenesis phenotype of the host. Scientists continue to identify the many molecular lesions that can lead to cancer progression and recognize that each tumor harbors its own genomic signature.[3] The basic question that remains to be answered is which part(s) of the molecular signature are related to the primary oncogenic event, and which are secondary.

The traditional picture of a linear evolution of a cancer through clonal expansion driven by accumulation of sequential mutations inherent to the cancer clone has now been nuanced by the influence of tumor microenvironment. Most cancers are a mixture of cancer cells and normal host cells that have been recruited to the site, or that have been induced to action by oncogenic changes occurring in cancer cells during malignant transformation. In genetically complex forms of cancers, it is difficult to define a specific “driver gene” within the multiplicity of gene alterations, unless one can evaluate the quorum of signals within the tumor microenvironment. A vastly improved ability to establish the hierarchy of genomic alterations present in the tumors of individual patients will be needed for a correct analysis and interpretation of biological information.

Despite the incomplete and continuously amended molecular information, and notwithstanding the fragmented understanding of its usefulness for effective anti-cancer therapies, many molecularly-based therapies have been implemented with spectacular success. Yet, as the example of imatinib demonstrates, the deployment of targeted therapy - from its discovery to standard of practice clinical use - can take more than thirty years in the present clinical climate.[4] Even in the case of CML, a cancer with a single therapeutic target, the traditional route to clinical implementation of *bcr/abl* complex inhibitors was uncomfortably slow. The process may be streamlined in rare diseases - the use of denosumab (inhibitor of RANKL) for the treatment of giant cell tumor of the bone - but the implementation of even a single agent therapy is filled with trepidations and insurance denials. It is therefore not surprising that for those diseases with activation of more than one molecular pathway, the implementation of molecularly-guided therapy remains challenging.

Therapeutic strategies incorporating inhibition of multiple molecular pathways will need to address the considerable differences in tumors between individuals, the heterogeneity within a single tumor, as well as the differences between the primary tumor and its metastatic lesions. Numerous and quite comprehensive catalogues of somatic mutations obtained by comparing a patient's tumor DNA/RNA sequences to his/her germline DNA/RNA[5, 6] indicate a great deal of heterogeneity in cancer genome evolution across different tumor types, across individual patients with the same tumor type, and even within a tumor.[7, 8] Considering this heterogeneity, the present appeal of enhancing the traditional site- and histology-specific treatment protocols with a more personalized approach (ie. precision medicine), can be more easily understood.

Scientists[9, 10] and leading politicians[11] have recognized that supporting progress toward precision medicine and increasing the use of biological therapies holds a strong promise of not only improving health outcomes,[12] but also of potentially improving cost effectiveness of cancer therapies.[13] The concept of precision medicine, as heretical as it may have initially sounded in cancer therapy, is not foreign in medicine. We test for antibiotic sensitivity, and we match blood for HLA subtypes in transfusion and transplantation medicine, and it is not surprising that our cancer patients are beginning to demand the same.[14] Ultimately, effective, precise, target-tailored medicines may abolish the use of old-fashioned cytotoxic treatments, or at least eliminate the need for maximum tolerated doses of radiation and chemotherapy. The implementation of these new treatment modalities will, require a number of necessary changes to the oncological practice and research in oncology. We will need to:

1 change clinical trial design in order to obtain efficacy data from *n* - 1 trials
2 provide and interpret large data while maintaining excellent data integrity
3 develop novel mathematical approaches for establishing hierarchy of genomic alterations in individual tumor samples
4 provide combination therapies based on pathway analyses
5 avoid combinations with maximum tolerated doses of chemotherapy: the argument for low dose (metronomic) chemotherapy backbone

## THE NEED TO CHANGE CLINICAL TRIAL DESIGN IN ORDER TO OBTAIN EFFICACY DATA FROM N - 1 TRIALS

Medical practice is a conservative vocation, and one of the most often repeated quotation in medical lore is: *Primum non nocere* (“first do no harm”). As such, in order to facilitate the translation of precision medicine to practice, sufficient evidence about precision medicine being as good or better than present therapies is requisite for the larger scientific and medical community to use the therapy. Unfortunately, over the last 40 years various regulations, were instituted in order to protect the public from unfounded claims of cure. While these were initially created for the benefit of the patient, they have led to a very inflexible structure of clinical trials - one that is no longer optimal for testing of new biological agents. Present clinical trials involve the addition of a single new agent to standard, established, maximum tolerated dose of therapy. To arrive at such a trial, the new agent must first go through a dose finding (dose escalating) trial (Phase I), which determines its maximum tolerated dose (MTD). The need to know the MTD is based on the well ingrained notion that the relationship between dose and cancer cell kill is linear[15] and more must be better. The notion, even though disavowed by the same scientist that first introduced it[16, 17], continues to be very dominant in oncology, even though some oncologists have begun using lower doses of chemotherapy in combination with targeted therapies.[18–21]

**Figure 1:**
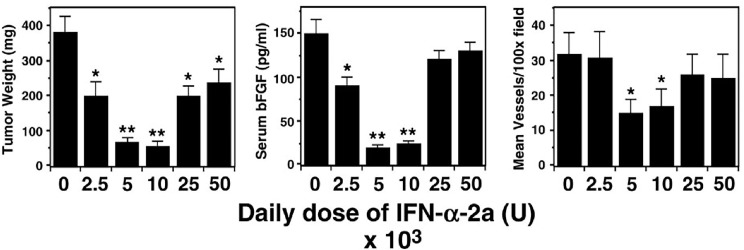

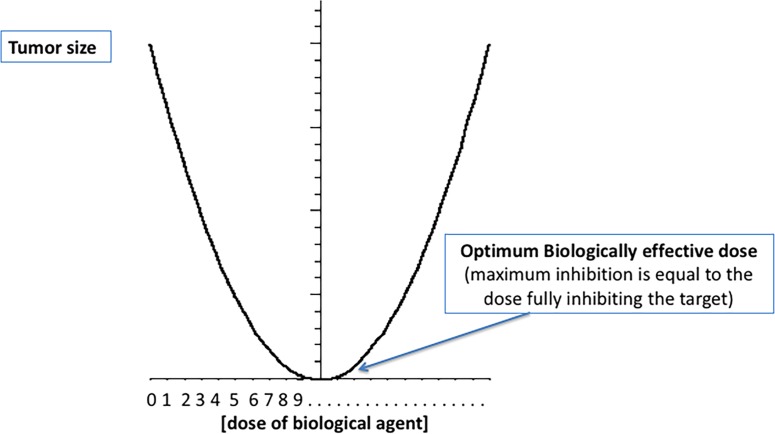
The U-shaped curve associate with the effect of biological therapies Unlike the linear relationship between dose and cell kill assumed in the early work of Skipper and Schabel^15^ - the effect of a biologic agent may differ at low and high doses. Panel A is an adaptation of figure first published by Slaton^23^ in 1999. The optimum biologically effective dose is often a medium rather than maximum dose. This U-SHAPED CURVE may facilitate the initial up and the subsequent down-regulation during physiological biological processes. In a stress response a linear increase of interleukins is desired during the initial stress, but a relaxation needs to follow in presence of excess ligand.

Once the MTD is defined in Phase I trial, the agent is put through an early efficacy trial (Phase II), before proceeding to a randomized, double blind, placebo-controlled (Phase III) trial to validate its efficacy, and to post-marketing surveillance studies (Phase IV). While Phase I-IV trials were informative for evaluation of the conventional surgery/chemotherapy/radiation approach, it is not optimal for biological agents where optimal dose is not the MTD and where toxicities are minimal.[22] This particular point is further discussed in section 5.1, and represented graphically in the Figure 1. Phase I-IV clinical trial design may not only be unsuitable for testing biological agents, they may be detrimental to the testing of biologically based therapies because most biologic agents sensitize to chemotherapy and radiation, and thus heighten the toxicity in the combination arms.[23, 24]

A body of pre-clinical and clinical evidence indeed suggests that the relationship between the dose of a biologic agent and its effect is NOT linear.[25, 26] It is most commonly U-shaped. One of the earliest publications suggesting this phenomenon showed that the effect of interferon alpha 2B differed at low, medium and high doses[27] (see Figure 1A). This was subsequently found to be true for most biologic agents, especially those that depend on receptor/ligand interaction. Once all receptors are engaged, and the full effect achieved, any further increase in dose leads to off-target effects rather than further receptor inhibition. The excess of drug therefore intensifies toxicities. For example, while the effect of TGF beta1 at low doses is anti-tumorigenic, its effect at higher levels is pro-tumorigenic, creating a U-shaped response curve (see Figure 1B).[28] This characteristic u-shaped response curve of biological agents, termed hormesis,[26] further illustrates that levels and function of biological agents influence the equipoise of several pathways, and can be tumor suppressive or tumor promoting.

The doses of biological agents should therefore be determined by the optimal biologically effective dose, rather than by a maximum tolerated dose, and the Phase I/II trials are not suitable for the introduction of a biological agent to clinic. In the case of biologic agents more is not necessarily better, and dose escalations using the traditional Phase I trial may not only be inappropriate, they can be detrimental, because the effect of the biological agent at high doses may be opposite to the desired effect.[25, 26] The change in pharmacodynamics of metronomically dosed vinblastine vs MTD vinblastine provides a very good example. The dose of vinblastine used for inhibition of angiogenesis is many folds lower than the anti-proliferative dose of vinblastine (~6mg/m^2^).[29]

The fact that Phase I trials are in general meant to establish dose-limiting toxicities rather than offer therapy is something most patients may not be able to appreciate when a Phase I trial is presented to them as the “last option”. The chance of cure or even of a positive response is very small, especially *in situ*ations where the intended target is not tested for and may not even be present. While some early efficacy trials of targeted agents for relapsed cancers may show some effectiveness,[30] the response is rarely sustained.

The role of a randomized, double-blind placebo controlled trial (RCT) is similarly questionable in an era where precision medicine is available. An RCT is in principle a comparison of two populations, one with and the other without the tested agent. Its goal is to find an agent that would be effective for the largest percentage of the general population, rather than optimize therapy for an individual. Because identifying the best treatment for an individual is so fundamentally different from a treatment that performs best at the population level, it is highly unlikely that Phase III approaches will be able to capture the outcomes of targeted therapies in precision medicine.

There is an early level of recognition of the need to revise the present model of clinical trials. Timely changes to clinical practice have been suggested by the recent National Cancer Institute Precision Medicine Initiatives for the new National Clinical Trials Network,[31] but most molecular testing continues to be used only as means to streamline the enrollment in clinical trials. In order to accommodate the *n* = 1 trial model, early discussions have been initiated about creation of a “cancer knowledge network”,[10] where information from the numerous case studies of truly individualized cancer treatments could be shared and evaluated. A case in point is the early effort to collect data from patients using targeted therapies in the NCI-Molecular Analysis for Therapy Choice (NCI-MATCH) Trial. In this trial, which opened in August 2015, analyzes patients' tumors to determine whether they contain genetic abnormalities for which a targeted drug exists (that is, “actionable mutations”) and assigns the patient to a clinical trial based on one of the detected abnormalities. While the trial will make some data available, its limitation lies in its traditional trial design. The trial suffers from two shortcomings; one, it is likely that of the hundreds of patients tested, only very few will find a matching clinical trial, and two, even though the tumor tissues will be analyzed for more than 4,000 different variants across 143 genes, patients with more than one genomic abnormality will still be enrolled on a single agent therapy trial, ignoring the actual tumor biology. This approach does not change the paradigm, as it does not address the complexity of tumor biology, heterogeneity and especially not the need for pathway analysis in cancer therapy.

A special problem in clinical studies is the current practice to include at first instance only relapsed and refractory patients. As mentioned, malignant cell proliferation is under control of the primary oncogenic event, but secondary (acquired) changes may independently control further malignant cell proliferation. The chance that analysis of tumors in newly diagnosed patients may elucidate the basic oncogenic driver(s) and the respective pathway(s) is much more likely. In this respect, newly diagnosed patients with cancers where the prognosis is poor should be considered for individualized therapies before resorting to the present standards. In children with poor prognosis disease, a well designed up-front window therapy, would clarify response to biological agent(s) more clearly. Examples where these studies should be considered are children with metastatic sarcoma, brain tumors or neuroblastoma where up 80% of children die despite elaborate standard chemotherapy and radiation protocols. To identify the basic oncogenic driver(s), all newly diagnosed malignancies would need additional molecular analysis as mentioned below.

## A POTENTIAL SOLUTION

**Figure 2:**
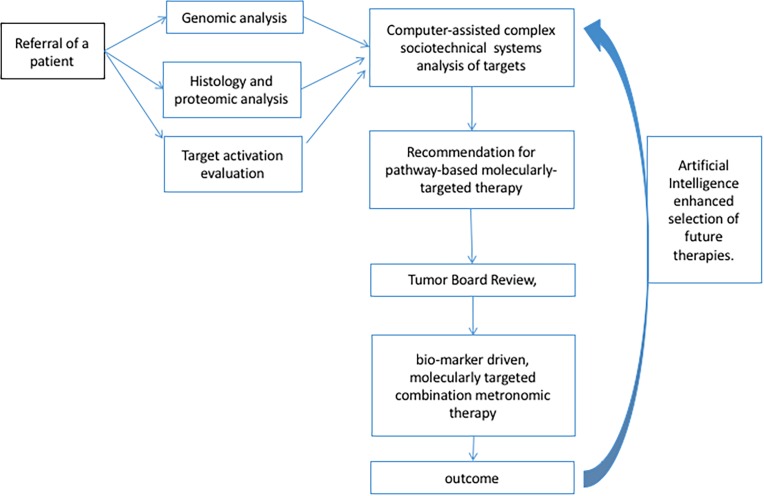
Pathway to combination targeted therapy design The ability to evaluate outcomes of combination targeted therapies is dependent on the ability to standardize selection of therapeutic targets and low-dose metronomic backbones. The diagnosis of patient's molecular profile should be based not only on the genomic analysis of the patient's and the patient, but also on detecting the target proteins and their activation in the tissues. In order to incorporate, and consolidate the vast amount of information computer-assisted complex sociotechnical systems need to be employed to provide tumor boards with up-to-date information about the best molecular targets. Finally, to continuously improve the quality of the information provided to tumor boards, AI should be used to inform future decisions.

To remedy the difficulty of collecting individual case study data we propose formation of consortium(s) of pediatric and adult institutions providing a standardized approach to selection of targets aided by computer assisted information processing and facilitated through an online tumor board review. The outcomes of the individual cases within the consortium(s) can then be pooled, evaluated, and used to inform selection of targets for future patients in real time (Figure 2). It is unlikely that all collaborative groups will be able to use the same tissue biomarker analysis outside a collaborative clinical trial. Only a collaborative, synchronized evaluation can lead to the meticulous collection and sharing of the DNA/RNA/Protein tissue analysis, that can lead to standardized selection of targets and therapeutic agent combinations, and where meticulous collection of the respective outcomes can be done.

The approach of this consortium has some similarities to the efforts extended by the ECOG-ACRIN Cancer Research Group, NMTRC, SWOG, Alliance for Clinical Trials in Oncology, NRG Oncology Group and the multiple sites participating in the NCI National Clinical Trial Network for establishing the MATCH trial. But it differs, in its use of using bio-marker driven, molecularly-targeted metronomic combination therapy. The consortium(s) stresses the use of a multi-target, multi-modality approach rather than enrollment on single agent trials. The hope is that sufficient amount of data will be accumulated to provide the necessary evidence to inspire other organizations to extend the examination of tumor tissue to include genomic, proteomic and metabolomics examination of the host as well as of the tumor, and promote individualized cancer therapies. Because only a very small number of patients is going to have overlapping molecular alterations and as such require the same combination of agents, traditional population-based statistical approaches comparing two disparately treated groups may not be applicable, and novel statistical approaches using predictive models of cancer growth are going to be needed. The data from all individual patients treated by a precision medicine approach will be stored in a single de-identified database to be shared not only with the consortium members but also with other clinicians and researchers interested in using targeted approaches.

The additional benefit of sharing information of these N = 1 trials is going to be learning about the changed pharmacokinetics as combinations of different agents are being used. Pharmacokinetic studies are an integral part of present PhaseI/IV clinical trial structure. If we remove this resource, alternative experimental procedures that would allow for establishing clearance and biodistribution of these biologic agents will be needed. We will need to provide the clinicians with means to be able to quickly identify the key factors that govern absorption, distribution, metabolism, and excretion of the individual biologics, [32] the pharmacogenomics, [33], as well as the effect of using combinations of agents. Consideration will need to be given to developing new intelligence-enabled tools for quick dose adjustments if more than one cyp3a4 or other members of the cytochrome P450 family involved in drug metabolism, are being used in the therapeutic regimen.

The information collected would, in addition to traditional outcome measures such as survival, response, and toxicities, include information about quality of life and health care costs. The outcome database could thus be used to not only inform future selection of therapeutic agents and their combinations based on response, survival and toxicities, but also aid in formulating fiscally responsible clinical strategies based on cost-effectiveness models.[13]

## THE NEED TO PROVIDE AND INTERPRET LARGE AMOUNTS OF DATA WHILE MAINTAINING EXCELLENT DATA INTEGRITY

However brilliant the physician may be, there is no way he/she is going to remember the millions of possible genetic variants and what each of those variants may mean for the individual patient. Moreover, given our continuously evolving understanding of the genomics, proteomics, metabolomics and other characteristics of tumor growth, it is unrealistic to expect any individual to remain current and on top of new discoveries. Invariably, in order for physicians to access and make use of the vast and constantly emerging information, she/he will need to use a variety of computational tools, and have access to a well-maintained computational support infrastructure. While initially, the focus of this computational infrastructure may be on tumor genomic signatures, and on genomic backgrounds of the hosts, it should eventually incorporate for a true personalized medicine application all of the patient's medical history, family history, dietary history, and exercise/activity information.

To implement precision medicine – and incorporate individual differences in genomic make-up and individual biological characteristics into treatment decisions – we will require the development and easy access to large-scale genomic, proteomic, biologic and health information databases. While some protein-protein interaction (PPI) networks are already publicly available on the Internet, they are, at least at present, mostly complex interaction maps developed by academic biologists over the last 50 years. Of concern is that because they are maintained by academic institutions with varied levels of funding, they may be of varied levels of information integrity, and of different ability to integrate emerging information or to provide for any corrections/additions driven by new information. Due to the clear and potentially immediate impact precision medicine can exert on cancer therapies much of the information in these databases are dedicated to oncology. However, the long term goal should be to generate a broad ranging source of information about diseased and physiologic states that would be useable for general medical purposes.

## A POTENTIAL SOLUTION

To address the difficulty accessing, curating and interpreting large data, a clinician-relevant computer assisted search of available information of the publicly available databases needs to be created. While more information than ever is available to the clinician, the information is not only overwhelming, it is also dispersed across varied and copious sources, few of which are geared to clinical applications. Automated systems that can trawl, collect and align available relevant information and provide assistive interpretations for clinicians would significantly alleviate this problem. We can begin by accessing available information in publicly available academically or National Institute of Health curated databases and incorporate cancer knowledge networks as they become available. Such augmented human intelligence can improve the ability of an institutional tumor board to understand and interpret all of the available gamut of molecular information and remaining current on published medical information.

The computerized system, containing a variety of artificial intelligence technologies can integrate a wide variety of information and apply an “understanding” of cancer biology in order to guide a tumor board in designing the most effective therapy for each of its unique patients. The system can do so by incorporating and cross-referencing information from multiple modalities, integrating this information in a clinical oncology context, and providing mathematical analysis of molecular pathways relevant to the patient's specific (identified) molecular changes. The information incorporated into this stream can come not only from traditional academically curated databases, but also from medical and popular scientific literature sources, public media as well as health/fitness tracking databases as recovered through social media. The information relevant to the individual patient can therefore superimposed onto a consolidated and highly cross-referenced informational stream providing the safest avenue for using the most up-to-date and continuously extended by emerging information.

## THE NEED TO DEVELOP NOVEL MATHEMATICAL APPROACHES FOR ESTABLISHING HIERARCHY OF GENOMIC ALTERATIONS IN INDIVIDUAL TUMOR SAMPLES

While the advent of genomic testing - whether by a panel of genes or the entire genome - offers tremendous potential in clinical decision-making. There is presently a dearth of choices in ways to interpret and apply the information to the clinic. Scientists and clinicians are besieged with methods for differentiating between *driver genes* and *passenger genes*, realizing that not all gene alterations detected in cancer tissues are of equal importance. The conservative approach has been to use an expert-approved panel of candidate oncogenes and tumor suppressor genes in clinical testing. However, most candidate gene panels test only for gene alterations well documented in the literature and other authoritative sources. Those targets are ‘assumed’ by experts to be necessary for cancer progression based on the fact that some of these candidate genes have been around for decades. They may be considered universal driver genes just by virtue of our familiarity with them and their commonness. While these candidate approaches help alleviate the information glut, they are based on insufficient information given our relative paucity and incomplete knowledge about the role genetic mutations may play in the host, in tumor specific host tissues, and/or in cancer biology. While BRAF^V600E^ and BRAF^V600K^ mutations are established driver genes for neuroectodermal tumors such as melanoma, the use of BRAF fusions, and non BRAF^V600E^ or non BRAF^V600K^ gene alterations in gliomas will have to be established.[24, 34]

To use and organize the continuously emerging and heterogeneous information being deposited into genomic (The Cancer Genome Atlas, TCGA; Gene Expression Omnibus, GEO; the NCI's Database of Genomic Structural Variation; dbVar etc), proteomic (UniProt, Swiss-Prot end may others), and metabolomics (Kyoto Encyclopedia of Genes and Genomes, KEGG; and other) databases, as well as the concerted effort to identify and catalog genomic vulnerabilities across hundreds of cancer cell lines (Broad Institute's Project Achilles), new computational tools for repeated and potentially automated analysis of large data sets need to be developed.

## A POTENTIAL SOLUTION

The impetus lies in improving the ability to select the most appropriate therapeutic target(s) for a particular patient. This necessitates development of novel approaches for large genomic or proteomic data analysis through multidisciplinary collaborations between mathematicians, physicists, statisticians, pharmacists, physicians, bioinformaticians, artificial intelligence developers, biologists and software developers. The trans disciplinary process is mandatory in order to cover the end-to-end process, from cancer diagnosis, to testing for genomic alterations, to selecting appropriate targets, to analyzing pathways involved in cancer progression, to the design and administration of therapies. The motivation should be improving the ability to select the most appropriate therapeutic target(s) for a particular patient.

There are two approaches to this. The first is more established and uses high-throughput statistical analysis (bioinformatics) of genomic data such as mRNA transcriptomes or RNA Seq from tumors of a population of patients with the same disease.[35–39] This approach provides the means to identify the most frequent genetic alterations in a population. The alternative approach applies novel mathematical and physical methods to determine how the individual patient compares to the genomic information derived from the population studies.[40, 41] While it is expected that both approaches will merge in the not too distant future, they remain distinct at present and exist in two separate solitudes. Yet, in order to base a treatment decision on the unique molecular signature of the patient's tumor, an a priori resolution of the detected molecular alterations using both methods is an absolute starting point for the process.

One previously described novel physical method for prioritization of targets applies a thermodynamic interpretation to gene expression, and then uses a topological filter to identify a set of potential therapeutic targets by their predicted effect on survival.[42, 43] The method makes use of publically available protein-protein interaction networks (PPI networks). These PPIs are online repositories of interaction datasets compiled by international teams of academicians and researchers, and comprehensively curated into networks akin to telecommunication or social network maps. The thermodynamic entropy method considers these PPI networks a closed system where all interactions tend to equilibrium, and where entropy is a measure of the PPI network disorder. Because degree entropy of PPI networks for different cancers, correlates with likelihood of survival of patients with this cancer,[43] one can calculate the effect of eliminating a specific target (or eliminating multiple targets). This approach has demonstrated promising results, and points to the benefits arising from incorporating multidisciplinary perspectives to cancer models.

Another previously described method performs a pan-cancer analysis of mutated networks.[44] This unbiased and open-ended analysis had revealed 16 significantly mutated subnetworks that were not previously thought to play significant role in cancer, and demonstrated that rare combinations of mutations, across multiple PPI networks may provide new insights and new opportunities for diagnostics and therapeutics across cancer types.

The PPI approach can be used in a number of ways. For instance, one can overlay transcriptional data from a single patient onto a PPI network, or a data set from The Cancer Genome Atlas (TCGA). As an example of the later, TCGA transcription data from a population of patients with glioblastoma multiforme (GBM) was overlaid on the BioGrid PPI network. The current Biogrid Index[45, 46] version 3.3.124 (http://thebiogrid.org/), holds more than 820,000 protein interactions derived from high-throughput datasets, individual focused experiments, and from over 44,000 publications. The types of protein-protein interactions include actual chemical bonding, or temporary bonds known as secondary bonding, and the concentration of the specific proteins dictates the degree of interaction. If a protein is in limited supply, it is said to have low chemical potential, and if it is abundant, it is said to have high chemical potential. Thus, using protein concentration, we can calculate the chemical potential of each protein in the network (i.e. Gibbs free energy), compute a topological measure known as filtration threshold (an energy threshold), and “filter out” the most energetic subnetworks from the larger network and try to reduce complexity of these subnetworks by inhibiting each protein in turn. Using this strategy, the “best therapeutic targets” are those that, when inhibited, most effectively reduce the complexity of a PPI network.

As an alternative, one can superimpose patient-specific tumor mRNA transcription data (a surrogate for protein concentration) onto BioGrid, calculate Gibbs free energy for all proteins in the network, and identify those nodes with most effect on entropy. Many of these nodes may not have been identified in the specific tumor type. For example, BRACA1, an accepted therapeutic target in breast or ovarian cancer, was identified as best therapeutic target for 41 out of 342 glioblastoma multiforme (GBM) patients in TCGA,[47] even though the importance of its overexpression in GBM is unknown. Similarly SIN3 was important in 38 of the 342 GBM patients in TCGA, and SIN3 turns out to be a member of a regulatory complex in the biology of glioblastoma.[48] A total of 46 unique targets were identified using GBM transcription data from 342 patients with glioma available in TCGA.

The complex sociotechnical system[49] considered here should be designed to work with as much genetic, proteomic and biologic information as available, and involve as many fields of expertise as possible. It should be noted, that even though it is being designed for maximum efficacy in cancer (both solid tumors and leukemias/lymphomas), it can be broadened to cardiology, inflammatory bowel disease and other medical specialties as genomic information in these fields emerges. It is able to use full transcription information from the tumor tissue; subtractive transcription information of tumor tissue and patient normal tissue; proteomic analysis of the same; phosphorylation maps, methylation arrays etc. At a minimum, it requires genetic information in the form of gene expression (transcription) microarrays or a panel of genes. Its strength lies in being able to continuously incorporate new information, as well as new mathematical and thermodynamic methods for therapeutic target prediction.

## THE NEED TO PROVIDE COMBINATION THERAPIES BASED ON PATHWAY ANALYSIS

Treatment decisions are, at least in present oncology practice, made on the basis of histological diagnosis, site of tumor origin (breast, lung, prostate etc), and the familiarity of the oncologist with a therapeutic agent. Despite the documented genetic and biological differences in even histologically identical site-specific cancer types,[8] most first line therapies do not diverge from the National Comprehensive Cancer Network (NCCN) Guidelines for Treatment of Cancer and national guidelines in other countries by site. They do not incorporate RNA/DNA sequence, transcription or protein expression information. Despite the evidence that molecular signatures of seemingly diverse and distinct cancers (lung squamous, head and neck, and a subset of bladder cancers) can coalesce into a common, site-independent molecular subtype,[50] most patients are still treated according to cancer site specific protocols. If considered, new treatment modalities are used only in second or later line of therapy, when additional molecular changes may have been added to the cancer initiating event adding to the complexity of controlling cancer growth.

It is encouraging, however, that more and more oncologists are looking for safe and rational ways to incorporate genomic and biological information into first line therapies and individualize treatment protocols. This is especially true for oncologists treating patients with poor prognoses cancers such as sarcomas or brain tumors. But the approaches differ widely. The phrase “precision medicine” or “targeted therapies” are employed to describe a wide range of approaches in clinical oncology such as:

1 Targeted therapies used because a specific, single molecule is presumed to be present on the basis of previously published data (populational approach).
2 Therapies where, based on the histology of the tumor, a specific molecular target is looked for, identified and, if the mutation is present, treated as part of a single agent trial (a candidate target approach).
3 Targeted therapies that test for a panel of candidate molecules (usually an expert established panel of genes), but where a single target, selected either on the basis of its availability in a clinical trial, or on the availability of an FDA approved drug, is used (a panel of candidate targets approach).
4 Therapies that test the entire genome or transcriptome of the tumor and/or of the patient, but where a single molecular target is selected and treated.
5 Therapies that test the entire transcriptome and/or proteome and/or exome (note that the candidate approach is a subset of the full exome), a combination of molecular targets according to the ‘pathway activation strategy' is selected, and all targets contributing to tumor progression are treated (the position of the authors).

It should be stressed, that using targeted agents in absence of testing for molecular alterations may be detrimental.[12] A recent comparison of outcomes of patients treated with targeted agents without testing the tumor tissues for targets (i.e. non-personalized targeted therapies) was associated with significantly poorer outcomes than even traditional cytotoxic agents approaches.[12]. The same comprehensive analysis of phase II, single-agent arms revealed that, across malignancies, a personalized strategy was an independent predictor of better outcomes and fewer toxic deaths[12] Similarly, using strategies that do not use combination therapies and thus do not inhibit the majority of molecular pathways contributing to tumor progression (the single agent approach) also provide no benefit.[51] The SHIVA prospective randomized trial[51–53] compared a personalized approach with conventional therapy in relapsed refractory adult solid tumors. This was a single-agent treatment enrolling patients on the basis of limited molecular profiling of known targetable pathways, and it was not surprising that there was no difference in progression-free survival between the molecular alteration based therapy and conventional treatment. There may be more than one reason for the reduced efficacy of a single agent approach. There is a high likelihood of missing some important targets due to limited molecular profiling, and there is a high likelihood of treatment resistance due to alternative pathways with single agent approach. The use of several molecularly targeted agents in combination with low dose chemotherapy based on comprehensive analysis of individual tumor biology is an appealing way to counteract this type of treatment resistance.

The incorporation of tumor molecular signatures information into clinical practice has not been easy, and for most physicians the most acceptable manner of using tumor molecular signature information is to screen for commonly occurring alterations and to enroll the patient on a clinical trial using the particular inhibitor. While this may be a practical and rational solution, the approach is inadequate for patients with complex genomic signatures consisting of more than one gene alteration. With the exception of chronic myeloid leukemia (CML), gastrointestinal stromal tumor (GIST), dermatofibrosarcoma protruberans (DFSP), or other similarly rare cancers, single mutations rarely account for the complexity of cancer biology, or for the secondary gene activation(s) caused by alterations within the tumor microenvironment. The protection of cells from xenobiotic such as cytotoxic agents do not require a mutation, commonly an increased expression (or activation) of molecular pathways already encoded in the genome is sufficient for emergence of resistant clone. As such targeting a single gene alterations is unlikely to be effective in most tumors. As one pathway is inhibited, an alternate pathway is activated or additional genomic alterations are acquired.

A good example is provided in targeted treatment of melanoma using monotherapy. Treatment with either vemurafenib (BRAF inhibitor) or trametinib (MEK inhibitor) alone can lead to excellent, but invariably short-lasting responses [54, 55] due to feedback activation of other pathways.[56–58] Because most oncogenic changes tend to hijack physiologic host responses such as inflammation, nullify other host defense mechanisms such as immune surveillance, and/or re-activate dormant developmental pathways for angiogenesis, immune evasion, and growth – the feedback loops are endless. Because oncogenic BRAF^V600E^ can lead to melanoma cancer cell immune evasion,[59] and the reversal of this evasion by addition of PD1 or CTLA4 immunologic therapies has been shown to provide additional benefit to BRAF inhibition alone. The combination of immune checkpoint inhibitors and BRAF-targeted agents in melanoma suggests a synergistic action of these otherwise independent therapeutic modalities,[60, 61] and a much longer response duration. While there may be a specific genomic signature that corresponds to immune evasion,[62] the use of combination therapy using inhibitors of BRAF, MEK and immune checkpoint inhibitors has caused 2-year survival rates of patients with metastatic melanoma to rise to 79%.[63]

## A POTENTIAL SOLUTION

A potential solution to managing the information glut and helping the oncologist to provide patients with the right combination of targeted agents and chemotherapy, is to enable them to use all of the available information. While producing complete genomic, proteomic and metabolomics datasets for each patient is not feasible at present, it has been possible in some well-funded research units to access the entire tumor and host transcriptomic information. The more complete the information provided for the analysis of the involved pathway(s), the more complete the therapeutic coverage. Unfortunately, for most physicians practicing clinical oncology today, the most feasible option is using a panel of candidate genes, because this may be covered by the patient's insurance. At lease in the US, clinical ‘omic’ testing is restricted to genomic panels through CLIA certified laboratories. Even though this approach carries the inherent risk that some driver genes may not be identified, and thus not included in therapy, it is a good initiating step towards the future.

The complex sociotechnical system being deployed by the authors of this manuscript maps the available molecular information from patients' tumors onto an oncology interpretation knowledge base pooled and cross-referenced from multiple sources, and weighted in PPI networks according to the unique composition of the patient's distinctive molecular signature. The combination of genetic alterations and mutational variants are matched to a series of filtered (see above) PPI subnetworks corresponding to biologic pathways relevant to cancer growth and progression, thus identifying molecular lesions that can be targeted with therapeutic intent. This complex sociotechnical system then searches the available literature and other reliable resources to find therapeutic agents targeting the identified molecular lesion(s), and minimize the number of drugs needed to inhibit all pathways within the identified PPI subnetwork. The system also considers the topology and interaction of each of the identified anomalous pathways in order to use the minimum possible drugs, and still achieve the same therapeutic result. *in situ*ations where specific genomic alterations may confer an a priori resistance to a therapeutic agent,[64, 65] the agent is eliminated.

Roughly similar to the current use of Artificial Intelligence technologies deployed in recommending movies on the basis of our previous choices, likes or dislikes, one of the AI components in this system records and documents the selection of targets, the treatment protocols and the respective outcomes in order to inform future therapeutic selections. More specifically, as oncologists and other experts on the tumor board introduce novel evidence for, or arguments against a therapeutic choice provided by the system, the information is recorded and used to refine future pathway analyses. The hope is that genomic/proteomic information will become affordable and we will include the genomic/proteomic analysis as a standard component of the electronic medical record. In turn, as more information from patient's medical record is incorporated, we will be able to consider any co-morbid conditions of the host and filter out harmful or ineffective drugs from the therapy recommendations further, resulting in improvement of the safety of our treatments.

## THE NEED TO AVOID COMBINATIONS WITH MAXIMUM TOLERATED DOSES OF CHEMOTHERAPY: THE ARGUMENT FOR LOW DOSE (METRONOMIC) CHEMOTHERAPY BACKBONE

A commonly employed approach for enhancing the ability chemotherapy to fight cancer is to use chemotherapy in combination with a biological agent. An assumption is made that the inhibitory effect of the biological agent would be additive to the effect achieved by traditional chemotherapy or radiation. However, the use of biologic agents, especially those inhibiting host responses (such as angiogenesis or inflammation), strip the anomalous cells (but also the patient's normal cells) of its defense mechanisms such as growth factors and inflammatory cytokines and lead to sensitization of all cells to DNA damaging agents such as radiation or chemotherapy. Because most mechanisms used to protect cells from xenobiota such as chemotherapy or radiation tend to activate developmental pathways already encoded in the genome, inhibition of these pathways increases toxicities whenever standard (maximum tolerated) doses of chemotherapy or radiation are used with biological agents.[66]

In a standard clinical trial, where a standard arm is compared to standard arm with the biological agent, the approach greatly disadvantages the intervention arm. The combination of the biologic agent and high dose chemotherapy, makes an already maximally toxic regimen lethal. As a result, the benefit of any tumor response will be concealed by these increased toxicities, and no overall survival benefit will be seen.[66] An example of this is the case of combining bevacizumab with standard MTD chemotherapy. While the RIBBON2 trial showed an improved progression-free survival compared to patients treated only with chemotherapy alone [PPS 7.2 months in the experimental group compared to 5.1 months in the chemotherapy only arm (*p* − .0072)]. The 10% improvement in overall survival rate was not statistically significant.[67] Based on this finding, the US Food and Drug Administration (FDA) revoked the approval of bevacizumab as a first line treatment for breast cancer, even though the majority of women had responded, and some remain well controlled on the drug to date.

The concept of “metronomic chemotherapy” was initially introduced in the year 2000,[29, 68, 69] and constituted a marked departure from the classic model of maximum tolerated dose (MTD) strategy. It emerged in the face of early clinical and pre-clinical evidence supporting its ability to suppress tumor growth even in cases where the cancer cell was resistant to the MTD of the used chemotherapeutic agent.[29, 68, 70, 71] Unfortunately, the concepts were poorly understood and underused. It has gained momentum however and at present it is being adopted with increasing frequency around the world,[72–74] and the website www.clinicaltrials.org now lists over 150 trials that use the word “metronomic” in their title. The mechanism of action of metronomic chemotherapy has been subject to excellent recent reviews,[75] and its value to implementation of precision medicine well documented.[22] To summarize briefly, because of the side effects induced by maximally dosed chemotherapy, the duration of the therapy has to be limited and breaks for bone marrow recovery incorporated. Furthermore, because conventional chemotherapy targets only proliferating malignant cells, a large portion of malignant cells is not affected. Only once these cells are re-engaged in the cell cycle process cytotoxic drugs are able to corrode them. Metronomic therapy implies that the use of low, continuous doses of chemotherapy in combination with biologic response modifiers not only avoid toxic side effects, but also preferentially target the host biological responses such as stromal induction,[76] angiogenesis,[68, 77, 78] immune surveillance,[75, 79, 80] and inflammation.[76] Angiogenesis and inflammation represent a physiological repair mechanisms hijacked by the proliferating tumor and actively contributing to tumor cell re-growth. The enormous success in the treatment of pediatric acute lymphoblastic leukemia, is at least partially due to the one and a half year long maintenance low dose metronomic chemotherapy.

Thus, it should be stated that in cases where up-front eradication of the cancer is not possible with MTDs, the MTD-induced up regulation of host inflammatory responses, rather than defending us from cancer, contributes to subsequent cancer progression. Because MTD chemotherapy kills only chemo-sensitive cells with each cycle, the chance of selection of a chemotherapy resistant subpopulation and recurrence is very high.

**Figure 3:**
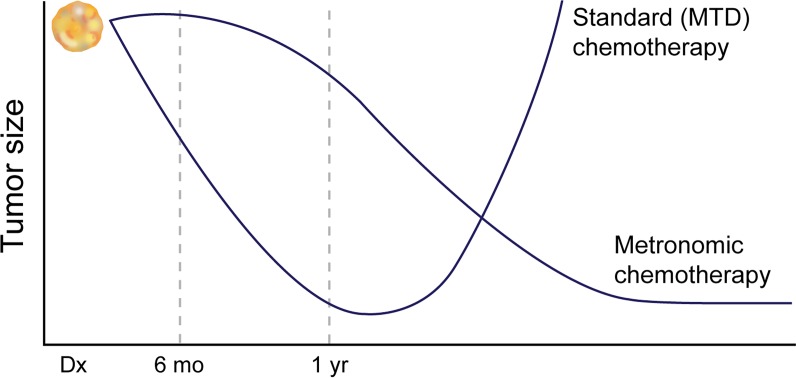
Comparison of Metronomic and Standard dose strategies The onset of action of metronomic chemotherapy is slower, but because of its ability to suppress biological processes such as angiogenesis or inflammation which are often “hijacked” by the tumor for growth, and because it avoids selection of the resistant population of cells, its effects are more sustained. However if comparison of these two therapies is made before 6 months, the wrong conclusion about the effectiveness of metronomic chemotherapy may be made.

Metronomic chemotherapy, with its goal of long-term “tumor control”, lower toxicity, and prevention of tumor progression (rather than immediate reduction in tumor size), may represent a more realistic strategy for cancer therapy. This is especially true for cancers not amenable to upfront cancer eradication. While slower in its onset of action (see Figure 3), metronomic dosing has demonstrated better long term tumor control, even for cancers rendered resistant to the same drug under MTD,[29, 68, 78] because the low-dose chemotherapy approach avoids selection of a resistant cancer cell population.

A very strong argument for the use of a metronomic chemotherapeutic backbone in combination with targeted therapies is the risk of metastatic growth.[81, 82] This risk of exacerbating metastases has however, only been documented with single agent therapy and only in pre-clinical murine models. It remains theoretical in clinical practice where it is usually prevented by the synergistic action of biologic agents and low dose chemotherapy. The same is true for avoiding emergence of therapeutic resistance with targeted agents alone.[57]

## A POTENTIAL SOLUTION

In the coming decade(s) a background for the combination therapies will be applied for any patients with chemotherapy resistant cancer or for patients with very poor prognosis. As much information as possible should be gathered about the patient's tumor molecular signature, about the host specific germline gene alterations, and about the host phenotype as soon as possible, so as to avoid unnecessary toxicities and delays with standard therapies whenever success cannot be reasonable expectation. The hope is that data from each of these cases will be collected and each of the individual outcomes will inform any future therapeutic decision.

## CASE 1

A previously healthy 11 year old girl with neurofibromatosis type 1 was diagnosed in 2011 with a large right parietal glioblastoma multiforme following an episode of left sided weakness. She was found to have a hemorrhagic stroke, and despite a partial resection of the tumor, her hemiparesis never resolved. She was started on COG ACNS0822, randomized to Arm A, and she completed the 6 weeks of radiation and vorinostat. In November 2011 she started maintenance chemotherapy with Avastin 10mg/kg Day 1 and 14/Temozolomide 200mg/m2 Days 1-5 for 28 day cycle. She completed 11 out of 12 cycles before coming off protocol for disease progression in October 2012.

She was started on melatonin, metformin, cyclophosphamide and erlotinib based on a proteomic analysis done at Texas Children's. She progressed again within 2 months with leptomeningeal spread to the spine, and was changed to VP-16, vincristine, crizotinib, erlotinib, vorinostat. The regimen resulted in unacceptable toxicities with myelosupression, severe mucositis, and QTc prolongation with cardiac compromise.

**Figure 4:**
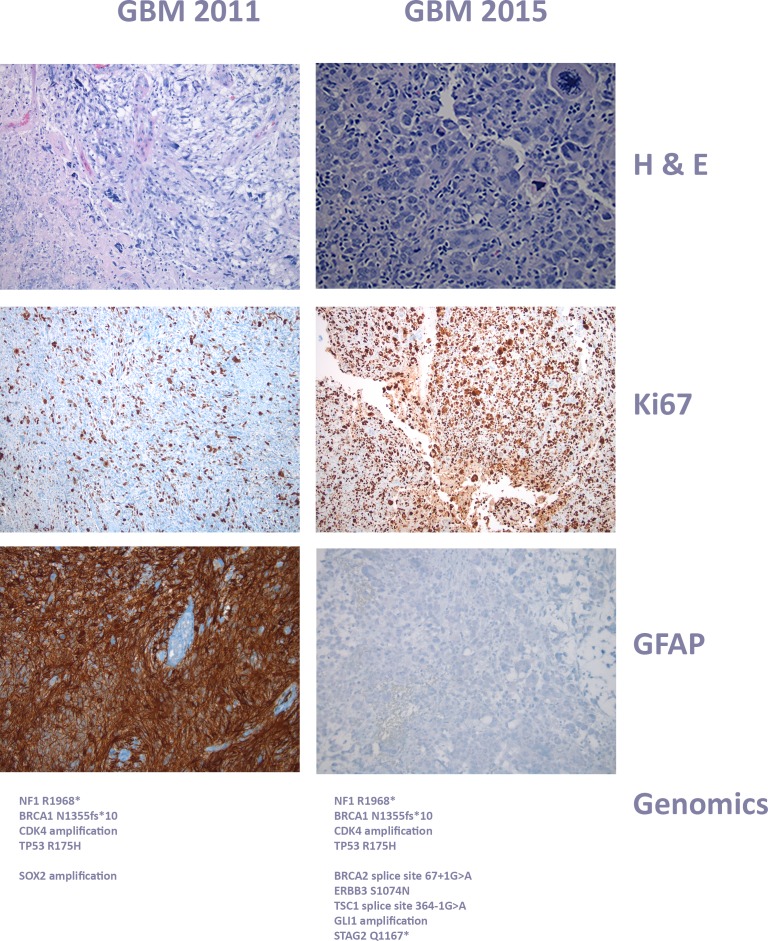
Histology of case 1, glioblastoma progression The original right temporal mass resected in 2011 showed glial neoplasm with vascular proliferation, necrosis, mitosis, and numerous pleomorphic cells, including rare giant cells. At this time, there was strong and diffuse immunopositivity for GFAP, and markedly elevated Ki-67 proliferative index, consistent with Glioblastoma WHO grade IV/IV. The original lesion regressed after the initial targeted therapy with sirolimus, sorafenib and metronomic VP16, but relapsed with a new extra axial lesion. The relapsed tissue in 2015 showed glial neoplasm with numerous tumor giant cell and atypical mitoe. The tumor cells were immunonegative for NEU-N, IDH-1(R132H) and BRAFv600E, but the molecular signature had obviously evolved, adding further genomic alterations. At this time, the tumor was negative for GFAP, and the ganglional component was no longer present. Both the 2011 and 2015 specimens had shown increased lymphocytic component and myxoid background, along with tumor giant cells, but the number of giant cells was increased significantly in the 2015 specimen.

She was taken off any disease directed therapy in March 2013 and referred to us for molecular analysis and individualized therapy. The characteristics of the tumor at diagnosis showed activation of a number of pathways associated with cancer growth and progression. The findings and initial pathology are summarized in Figure 4. The genomic analysis revealed NF1 R1968*, BRCA1 N1355fs*10, CDK4 amplification, TP53 R175H, SOX2 amplification. Because loss of neurofibromin function leads to increase in signaling through the Ras-Raf-MAPK and mTOR pathways, [83] she was started on sirolimus 2mg daily and sorafenib to inhibit growth factors downstream from these pathways in addition to metronomic (50 mg/m2) etoposide daily. She remained stable on this regimen until December 2015 (3 years) when she had a radiological progression.

She underwent an excisional biopsy and the molecular analysis of this relapse was consistent with a radiologically, histologically and genetically more aggressive phenotype (Figure 4). In addition to the original gene alterations, she now had BRCA2 splice site 67+1G > A, ERBB3 S1074N, TSC1 splice site 364-1G > A, GLI1 amplification, STAG2 Q1167*. Her therapy was therefore changed to everolimus (Ras-Raf-MAPK and mTOR pathways), ceritinib (GLI1/sonic Hedgehog pathway), and trametinib on a metronomic chemotherapy backbone of temozolomide 25 mg/m2, and remains stable.

The case provides a good illustration about the need for multi-agent therapy based on molecular signature. It also stresses the need to consider re-biopsy with relapse as the eco-evolutionary forces within the tumor microenvironment may cause therapeutic resistance and escape from tumor dormancy.[84]

## CASE 2

A 7-y old previously healthy boy with no family history of cancer was diagnosed with stage III abdominal Burkitt lymphoma in December 2014. He was initially treated standard BFM B-NHL 04 therapy, which included a single initial dose of 375mg/m2 Rituximab followed with 5 cycles of BNHL 04 chemotherapy consisting of dexamethasone, methotrexate, ifosfamide, cyclophosfamide, cytarabine, etoposide, doxorubicine, vincristine as well as intrathecal therapy. After 2 cycles, he had a very good partial response reaching < 5% of the initial tumor volume. An episode of the intestinal obstruction in February 2015 led to excision, and the histology confirmed sclerosing mesenteritis, without histological or rtPCR evidence of lymphoma (the original tissue was positive for cMYC translocation). The FDG PET was borderline positive, but this was thought to be due to inflammation.

Unfortunately the child was found to have an isolated radiological progression in the same region in which the intestinal obstruction had occurred two months after completing chemotherapy. The biopsy in June 2015 confirmed relapsed Burkitt lymphoma, this time with marked areas of sclerosing mesenteritis and mesenteric panniculitis. Mutational analysis of PI3K delta subunit proved germinal mutation/variant outside the classical Activated PI3K-delta syndrome (APDS) 1 or 2 variants. The mutational activation was confirmed by testing the patient's T- lymphocytes, and the S6 (Ser235/236) phosphorylation was found to be 33 fold that of a healthy control.

While undergoing the genomic testing, the boy was started on retrieval therapy with ibrutinib, obinutuzumab and ICE chemotherapy. Unfortunately, after a transient response and disease stabilization, he had an early progression following the first cycle. Based on the finding of germline mutation in PI3K delta subunit, he got 2 weeks of idelalisib (a phosphoinositide 3-kinase inhibitor, which blocks P110δ, the delta isoform of the enzyme phosphoinositide 3-kinase). The single agent therapy led to normalization of the S6 (Ser235/236) phosphorylation in patients peripheral T lymphocytes, but he had further disease progression. It was only when the combination of high dose cytarabine/etoposide (CyVe) with idelalisib and obinutuzumab was used that the disease was stabilized. A biopsy on 9/2015 showed a CD20 positive tumor, with high degree of proliferation and strong expression of PD-1L.

The second biopsy was analyzed using Affy GeneChip ST 1.0 and the whole transcriptome analysis confirmed increased levels of PI3K and revealed additional HR23B. Because HR23B can be used as a good predictor of response to HDAC inhibitors, valproic acid was being considered. Additional tumor specific (somatic) gene alterations in R273C and p53 were also shown.

The child, who had continued on oral ibrutinib + idelalisib and low dose cyclophosphamide since 9/2015, received palliative 21Gy local radiation. In 10/2015, based on the second biopsy findings, the nivolumab, a human IgG4 anti-PD-1 monoclonal antibody, and valproic acid, and HDAC inhibitor, were added. As of March 2016 the boy is doing very well. He has had partial response of the single residual abdominal tumor disease, and remains clinically well with Lansky score 100 and OS > 15 months. He comes to clinic biweekly for nivolumab infusions and assessments, but remains outpatient otherwise. He started his personalized therapy after his second relapse, and this 3rd EFS (7 months) is already the longest EFS, compared to 6 months post his initial standard BFM protocol and just 1 month post ibrutinib, obinutuzumab and ICE chemotherapy.

The case may illustrate a new variant of Activated PI3K-delta syndrome (APDS). At least at present the disease is not tested for and generally not recognized in children with Burkitt's lymphoma. Even if this child had a family history supporting testing for the autosomal dominant form of APDS, he would not have been found. Yet, he had an atypical germinal mutation in the gene that leads to lymphoid hyperplasia, and increases the risk of malignant transformation to B-cell lymphoma. The p110δ protein is a crucial subunit of the PI3K enzyme, and regulates activation of proliferative pathways in B-cells. As such, unless this constitutional activation can be blocked, it will be unlikely that 5 cycles of conventional chemotherapy could successfully prevent a relapse. It may be prudent, in cases where a mutational activation of an important proliferative pathway is found, to use maintenance biological therapy. This could be similar to the 2 years maintenance therapy used in childhood Acute Lymphoblastic Leukemia, which has cure rates of about 90%. It is our hope that this case illustrates a potential for keeping even children with poor prognosis due to genetically complex cancers at home. While not able to eradicate the cancer or it causative mutation, we may be able to keep them well, in school and active by prescribing a combination of low-dose metronomic chemotherapy, an immune checkpoint inhibitor, and a direct inhibitor of the activated pathway(s).

## SUMMARY

Many oncologists treating recurrent, chemotherapy resistant or poor prognosis cancers have begun re-purposing anti-inflammatory agents or immune modulators. Similarly, many oncologist use direct anticancer agents in an off-label setting to target specific genomic mutations regardless of the cancer subtype. An equal number of oncologists however, due to the time required for researching the vast amount of molecular information, continue treating children with conventional therapies. But for those cancers where the present chemotherapeutic, surgical and radiation strategies fail – the option of targeted strategies should be strongly considered.

The difficulty is that physicians using targeted therapies today do so without the benefit of computational infrastructure. While we use complex sociotechnical systems to manage nuclear plants and airports, we have not developed similar systems for the analysis and application of omics information. We need an efficient complex sociotechnical system that would allow us to analyze a molecular signature of the patient's cancer in minutes and select the appropriate molecular agent(s) in time for effective therapy.

We also need to abandon the present model of drug development. The present process often takes decades for each of the new therapeutic agents. Millions are spent testing each of the agents in individual Phase I-IV trials before its introduction to the clinic resulting in cost-prohibitive therapies. Most importantly however, thousands of patient lives are lost as we struggle to determine whether an agent “is clinically active” in the incorrectly designed clinical trials. A wealth of bioinformatics resources exists that can help narrow the choice of therapeutic combinations from the wide selection of already available molecular agents, and provide a treatment for a wide range of difficult to treat cancers TODAY.
